# Headache disorders and public ill-health in India: prevalence estimates in Karnataka State

**DOI:** 10.1186/s10194-015-0549-x

**Published:** 2015-07-22

**Authors:** Girish B Kulkarni, Girish N Rao, Gopalkrishna Gururaj, Lars J Stovner, Timothy J Steiner

**Affiliations:** Department of Neurology, National Institute of Mental Health and Neuro Sciences (NIMHANS), Bangalore, India; Department of Epidemiology, National Institute of Mental Health and Neuro Sciences (NIMHANS), Bangalore, India; Department of Neuroscience, Norwegian University of Science and Technology, Edvard Griegs Gate, Trondheim, NO-7491 Norway; Norwegian Advisory Unit on Headaches, Nevrosenteret Øst, St Olavs University Hospital, Trondheim, Norway; Division of Brain Sciences, Imperial College London, London, UK

**Keywords:** Headache disorders, Migraine, Tension-type headache, Medication-overuse headache, Epidemiology, Population-based study, Prevalence, Health policy, Global Campaign against Headache

## Abstract

**Background:**

Primary headache disorders are among the commonest disorders, affecting people in all countries. India appears to be no exception, although reliable epidemiological data on headache in this highly populous country are not available. Such information is needed for health-policy purposes. Our aim was to estimate the prevalence of each of the headache disorders of public-health importance, and examine their sociodemographic associations, in urban and rural populations of Karnataka, south India.

**Methods:**

In a door-to-door survey, 2,329 biologically unrelated adults (aged 18–65 years) were randomly sampled from urban (n = 1,226) and rural (n = 1,103) areas in and around Bangalore and interviewed by trained researchers using a pilot-tested, validated, structured questionnaire. ICHD-II diagnostic criteria were applied.

**Results:**

The observed 1-year prevalence of any headache was 63.9 %, with a female preponderance of 4:3. The age-standardised 1 year prevalence of migraine was 25.2 %; prevalence was higher among females than males (OR: 2.1 [1.7-2.6]) and among those from rural areas than urban (OR = 1.5 [1.3-1.8]). The age-standardized 1 year prevalence of TTH was 35.1 %, higher among younger people. The estimated prevalence of all headache on ≥15 days/month was 3.0 %; that of pMOH was 1.2 %, five-times greater among females than males and with a rural preponderance.

**Conclusions:**

There is a very high 1 year prevalence of migraine in south India (the mean global prevalence is estimated at 14.7 %). Explanations probably lie in cultural, lifestyle and/or environmental factors, although the observed associations with female gender and rural dwelling are usual. Levels of TTH, pMOH and other headache on ≥15 days/month are similar to global averages, while the very strong association of pMOH with female gender requires explanation. Until another study is conducted in the north of the country, these are the best data available for health policy in a population of over 1.2 billion people.

## Background

Headache is one of the commonest symptoms, and primary headache disorders are among the most ubiquitous disorders, affecting people in all countries [1]. India appears to be no exception [2]. The Global Burden of Disease Study 2010 (GBD2010) found tension-type headache (TTH) and migraine to be the 2^nd^ and 3^rd^ most prevalent disorders worldwide [3].

Nevertheless, knowledge of the prevalence of headache disorders, on which reiterations of the Global Burden of Disease Study depend, remains substantially incomplete. Historically, it has been gathered predominantly from the high-income countries of Western Europe and North America [1], leaving vast geographical areas almost data-free. Notable among these have been South East Asia, and in particular India. Neuroepidemiological studies in India have included headache only as one of multiple conditions of enquiry under the broad spectrum of neurological disorders [4]. There have been hospital-based studies of migraine [5, 6], but these do not provide information on prevalence or reveal the characteristics and impact of disorders in the population [7]. Yet India is home to over 16 % of the world’s inhabitants [8]. Lack of knowledge of the prevalence of and burden attributable to headache disorders among such a large community has an impact on the quality and meaning of global statistics [3]. In India itself, it stands in the way of effective health-care policy and planning, the delivery of services and the means of remedy.

Across the world, the knowledge gap is slowly being filled by a series of population-based studies supported by *Lifting The Burden* (LTB) [9, 10], a UK-registered nongovernmental organization conducting the Global Campaign against Headache [11] in official relations with the World Health Organization [12]. Methodology has been developed for this purpose [7, 13]. We undertook a population-based survey in southern India (Karnataka State) as part of this series. We focused on the headache disorders of public-health importance: migraine, TTH, medication-overuse headache (MOH) and other causes of headache occurring on ≥15 days/month. This paper describes the 1 year prevalence of these disorders in this population; subsequent papers will report headache-attributed burden.

## Methods

The methodology of the study has been published in detail previously [14] and is described only briefly here. The institutional ethics committee of the National Institute for Mental Health and Neuro-Science (NIMHANS) approved the study protocol. Informed consent was obtained from all participants.

The cross-sectional survey sampled from urban and rural areas in and around Bangalore: Kempegowdanagara, an urban administrative ward in the city of Bangalore, and Uyamballi and Doddaaladahalli, two large villages located 75–80 Km from Bangalore. A team of trained interviewers travelled to these communities and selected households through multistage cluster sampling. Interviewers called at each chosen household, listed all adult members (aged 18–65 years), selected one by simple random sampling and interviewed that person using a structured questionnaire. This instrument was an adaptation of the HARDSHIP questionnaire [13], translated into the local language (Kannada) in accordance with LTB’s translation protocol for hybrid documents [15]. It included demographic enquiry and a headache screening question (“Have you had headache during the last year?”) for all participants, followed by diagnostic questions based on the International Classification of Headache Disorders, 2^nd^ edition (ICHD-II) [16] and enquiries into burden for those reporting headache. Any participant reporting more than one headache type was asked to focus only on the one that was subjectively the most bothersome for purposes of description, diagnosis and prevalence counting. In the previously conducted validation study, the diagnostic part of the questionnaire had a specificity and sensitivity for migraine of 85 % (95 % CI: 81–89) and 63 % (52–72), and for TTH of 81 % (76–86) and 57 % (48–65) [14].

The survey continued in each community until the requisite sample (>1,000 biologically unrelated individuals from each stratum [urban and rural]) was achieved.

### Statistics and analyses

Data were entered into a secure database and statistical analyses performed using EPI INFO [17] and SPSS 15 [18].

Diagnoses were made not by the interviewers but by computerized algorithm [13] from the recorded survey responses. Participants reporting headache on ≥15 days/month were first separated, and described as a distinct group, with those also reporting regular use of acute headache medication on >10 days/month considered to have probable MOH (pMOH). To all others, the algorithm applied ICHD-II diagnostic criteria [16] in the order: migraine, TTH, probable migraine, probable TTH. Cases of migraine and probable migraine, and of TTH and probable TTH, were then combined for prevalence estimation and further analyses [7]. Remaining cases were unclassified.

We used proportions, 95 % confidence intervals (CIs), medians, means and standard deviations (SDs) to summarise the distributions of variables and chi-squared, Student’s t-test or ANOVA to test for significance of differences. We calculated odds ratios (ORs) to test for associations in bivariate analysis, and adjusted odds ratios (AORs) using multivariate logistic regression. We set the level of significance at 5 %.

## Results

There were 2,329 participants (1,141 [49.0 %] male, 1,188 [51.0 %] female, mean age 38.0 [±12.7] years, 1,103 [47.4 %] from rural areas and 1,226 [52.6 %] urban). The overall participation rate was 92.6 % (eligible population n = 2,514). While there were few actual refusals (25 urban, nil rural), the key reason for non-participation (103 urban, 57 rural) was unavailability for interview even after three contacts. The distributions of gender, age and habitation in the participating sample have been described, and were comparable to those of the population of Karnataka [14].

### Prevalence

The crude 1 year prevalence of any headache (n = 1,488) in the study population was 63.9 %, with female preponderance (73.0 % *versus* 54.4 % in males; OR = 2.3 [1.9-2.7]) and rural preponderance (71.2 % *versus* 57.3 % urban; OR = 1.8 [1.6-2.1]). Further analyses in this manuscript are by headache type.

The crude 1 year prevalence of migraine (n = 597) was 25.6 % (95 % CI: 23.9-27.4 %; 10.8 % [9.7-12.2 %] definite, 14.8 % [13.4-16.3 %] probable). Prevalence was higher among females (32.4 %) than males (18.6 %; OR = 2.1 [1.7-2.6]) and among those from rural areas (29.7 %) than urban (21.9 %; OR = 1.5 [1.3-1.8]) (Table 1).

**Table 1:** One-year prevalence of migraine by age, gender and habitation (N = 597)

Age (years)	One-year prevalence n (%) [95 % CI]								
	Urban habitation			Rural habitation			Total		
	Male	Female	Total	Male	Female	Total	Male	Female	Total
18-25	23 (17.6) [12.0-25.0]	27 (21.8) [15.4-29.8]	50 (19.6) [15.2-24.9]	16 (19.0) [12.1-28.7]	29 (25.9) [18.7-34.7]	45 (23.0) [17.6-29.3]	39 (18.1) [13.6-23.8]	56 (23.7) [18.7-29.5]	95 (21.1) [17.6-25.1]
26-35	32 (18.7) [13.6-25.2]	69 (32.5) [26.6-39.1]	101 (26.4) [22.2-31.0]	28 (20.6) [14.6-28.1]	63 (40.4) [33.0-48.2]	91 (31.2) [26.1-36.7]	60 (19.5) [15.5-24.3]	132 (35.9) [31.1-40.9]	192 (28.4) [25.2-32.0]
36-45	25 (15.2) [10.5-21.5]	51 (35.7) [28.3-43.8]	76 (24.8) [20.3-29.9]	42 (26.3) [20.0-33.6]	53 (41.1) [33.0-49.7]	95 (32.9) [27.7-38.5]	67 (20.7) [16.6-25.4]	104 (38.2) [32.7-44.1]	171 (28.7) [25.2-32.5]
46-55	13 (13.5) [8.1-21.8]	17 (24.6) [16.0-36.0]	30 (18.2) [13.0-24.8]	11 (14.7) [8.4-24.4]	36 (36.4) [27.6-46.2]	47 (27.0) [21.0-34.1]	24 (14.0) [9.6-20.0]	53 (31.5) [25.0-38.9]	77 (22.7) [18.6-27.5]
56-65	2 (4.1) [1.1-13.7]	10 (14.9) [8.3-25.3]	12 (10.3) [6.0-17.2]	20 (26.7) [18.0-37.6]	30 (39.0) [28.8-50.1]	50 (32.9) [25.9-40.7]	22 (17.7) [12.0-25.4]	40 (27.8) [21.1-35.6]	62 (23.1) [18.5-28.5]
All	95 (15.5) [12.9-18.6]	174 (28.3) [24.9-32]	269 (21.9) [19.7-24.3]	117 (22.1) [18.8-25.8]	211 (36.8) [33.0-40.8]	328 (29.7) [27.1-32.5]	212 (18.6) [16.4-20.9]	385 (32.4) [29.8-35.1]	597 (25.6) [23.9-27.4]

Table 1 shows migraine prevalence in the sample by age, which peaked in both genders in the range 35–45 years. A second rise was exhibited by males over 56 years, driven entirely by the rural population in which it was statistically significant (chi-squared = 18.99; *p* < 0.001); a small and statistically insignificant second peak was seen in rural females. The age-standardized 1 year prevalence of migraine (against Karnataka’s state population [8]) was 25.2 %.

The crude 1 year prevalence of TTH (n = 811) was 34.8 % (95 % CI: 32.9-36.8 %; 26.6 % [24.9-28.5 %] definite, 8.2 % [7.2-9.4 %] probable). Prevalence was similar between genders but higher among those from rural areas (38.4 %) than urban (32.2 %) (Table 2; chi-squared = 7.73; p < 0.005). TTH prevalence declined steadily from 40.1 % in those aged 18–25 years to 28.7 % in those over 56. This was reflected in both genders and in both urban and rural habitations (Table 2). The age-standardized [8] 1 year prevalence of TTH was 35.1 %.

**Table 2:** One-year prevalence of tension-type headache by age, gender and habitation (N = 811)

Age (years)	One-year prevalence n (%) [95 % CI]								
	Urban habitation			Rural habitation			Total		
	Male	Female	Total	Male	Female	Total	Male	Female	Total
18-25	44 (33.6) [26.1-42.0]	48 (38.7) [30.6-47.5]	92 (36.1) [30.4-42.1]	34 (40.5) [30.6–51.2]	55 (49.1) [40.0-58.2]	89 (45.4) [38.6-52.4]	78 (36.3) [30.1-42.9]	103 (43.6) [37.5-50.0]	181 (40.1) [35.7-44.7]
26-35	55 (32.2) [25.6-39.5]	68 (32.1) [26.2-38.6]	123 (32.1) [27.6-36.9]	54 (39.7) [31.9-48.1]	55 (35.3) [28.2-43.0]	109 (37.3) [32.0-43.0]	109 (35.5) [30.4-41.0]	123 (33.4) [28.8-38.4]	232 (34.4) [30.9-38.0]
36-45	55 (33.5) [26.8-41.1]	51 (35.7) [28.3-43.8]	106 (34.5) [29.4-40.0]	57 (35.6) [28.6-43.3]	48 (37.2) [29.4-48.2]	105 (36.3) [31.0-42.0]	112 (34.6) [29.6-39.9]	99 (36.4) [30.9-42.3]	211 (35.4) [31.7-39.3]
46-55	26 (27.1) [19.2-36.7]	20 (29.0) [19.6-40.6]	46 (27.9) [21.6-35.2]	26 (34.7) [24.9-45.9]	38 (38.4) [29.4-48.2]	64 (36.8) [30.0-44.2]	52 (30.4) [24.0-37.7]	58 (34.5) [27.8-42.0]	110 (32.4) [27.7-37.6]
56-65	11 (22.4) [13.0-35.9]	17 (25.7) [16.5-36.9]	28 (24.1) [17.3-32.7]	26 (34.7) [24.9-45.9]	23 (29.9) [20.8-40.8]	49 (32.2) [25.3-40.0]	37 (29.8) [22.5-38.4]	40 (27.8) [21.1-35.6]	77 (28.7) [23.6-34.4]
All	191 (31.0) [27.7-35.0]	204 (33.4) [29.6-37.0]	395 (32.2) [29.7-34.9]	197 (37.5) [33.2-41.4]	219 (38.9) [34.3-42.3]	416 (38.4) [34.9-40.6]	388 (34.0) [31.1-36.8]	423 (36.2) [32.9-38.4]	811 (35.1) [32.9-37.8]

There were 12 cases (0.5 %) of unclassified episodic headache.

The overall prevalence of all types of headache on ≥15 days/month (n = 68) was 3.0 % (95 % CI: 2.3-3.7). About 40 % of such cases were pMOH, of which the observed prevalence was 1.2 % (n = 28). While numbers were small, this disorder again showed a rural preponderance (1.5 % *versus* 0.9 % urban). More striking was the gender difference (Table 3): overall, prevalence was five times greater among females than males, while in urban areas pMOH appeared to be almost uniquely a disorder of females. There was not a clear age-relationship, but generally the highest prevalences were reported by those aged >56 years (4.2 % by females overall, and 4.5 % by urban females) (Table 3).

**Table 3:** One-year prevalence of probable medication-overuse headache by age, gender and habitation (N = 28)

Age (years)	One-year prevalence n (%) [95 % CI]								
	Urban habitation			Rural habitation			Total		
	Male	Female	Total	Male	Female	Total	Male	Female	Total
18-25	0 [0.0-2.8]	3 (2.4) [0.8-6.9]	3 (1.2) [0.4-3.4]	1 (1.2) [0.2-6.4]	2 (1.8) [0.5-6.3]	3 (1.5) [0.5-4.4]	1 (0.5) [0.1-2.6]	5 (2.1) [0.9-34.8]	6 (1.3) [0.6-2.9]
26-35	1 (0.6) [0.1-3.2]	1 (0.5) [0.1-2.6]	2 (0.5) [0.1-1.9]	1 (0.7) [0.1-4.0]	5 (3.2) [1.4-7.3]	6 (2.1) [0.9-4.4]	2 (0.7) [0.2-2.3]	6 (1.6) [0.7-3.5]	8 (1.2) [0.6-2.3]
36-45	0 [0.00-2.3]	1 (0.7) [0.1-3.9]	1 (0.3) [0.1-1.8]	0 [0.0-2.3]	1 (0.8) [0.1-4.3]	1 (0.3) [0.1-1.9]	0 [0.0-1.2]	2 (0.7) [0.2-2.6]	2 (0.3) [0.1-1.2]
46-55	0 [0.00-3.8]	2 (2.9) [0.8-9.9]	2 (1.2) [0.3-4.3]	0 [0.0-4.9]	3 (3.0) [1.0-8.5]	3 (1.7) [0.6-4.9]	0 [0.0-2.2]	5 (2.9) [1.3-6.8]	5 (1.5) [0.6-3.4]
56-65	0 [0.0-7.3]	3 (4.5) [1.5-12.4]	3 (2.6) [0.9-7.3]	1 (1.3) [0.2-7.2]	3 (3.9) [1.3-10.8]	4 (2.6) [1.0-6.6]	1 (0.8) [0.1-4.4]	6 (4.2) [1.9-8.8]	7 (2.6) [1.3-5.3]
All	1 (0.2) [0.0-0.9]	10 (1.6) [0.9-2.9]	11 (0.9) [0.5-1.6]	3 (0.6) [0.2-1.7]	14 (2.4) [1.5-4.1]	17 (1.5) [0.9-2.5]	4 (0.4) [0.1-0.9]	24 (2.0) [1.4-2.9]	28 (1.2) [0.8-1.7]

Among other types of headache on ≥15 days/month (overall prevalence 1.7 %; n = 40) there was a two-fold female preponderance but no apparent relationship with age or habitation (Table 4).

**Table 4:** One-year prevalence of other headache on ≥15 days/month by age, gender and habitation (N = 40)

Age (years)	One-year prevalence n (%) [95 % CI]								
	Urban habitation			Rural habitation			Total		
	Male	Female	Total	Male	Female	Total	Male	Female	Total
18-25	4 (3.1) [1.2-7.6]	2 (1.6) [0.4-5.7]	6 (2.4) [1.1-5.0]	1 (1.2) [0.2-6.4]	3 (2.7) [0.9-7.6]	4 (2.0) [0.8-5.1]	5 (2.3) [1.0-5.3]	5 (2.1) [0.9-4.9]	10 (2.2) [1.2-4.0]
26-35	0 [0.0-2.2]	5 (2.4) [1.0-5.4]	5 (1.3) [0.6-3.0]	0 [0.0-2.7]	4 (2.6) [1.0-6.4]	4 (1.4) [0.5-3.5]	0 [0.0-1.2]	9 (2.4) [1.3-4.6]	9 (1.3) [0.7-2.5]
36-45	3 (1.8) [0.6-5.2]	5 (3.5) [1.5-7.9]	8 (2.6) [1.3-5.1]	3 (1.9) [0.6-5.4]	3 (2.3) [0.8-6.6]	6 (2.1) [1.0-4.5]	6 (1.9) [0.9-3.9]	8 (2.9) [1.5-5.7]	14 (2.3) [1.4-3.9]
46-55	1 (1.0) [0.2-5.7]	1 (1.4) [0.2-7.7]	2 (1.2) [0.3-4.3]	1 (1.3) [0.2-7.2]	3 (3.0) [1.0-8.5]	4 (2.3) [0.9-5.8]	2 (1.2) [0.3-4.2]	4 (2.4) [0.9-5.9]	6 (1.8) [0.8-3.8]
56-65	0 [1.1-7.3]	0 [0.0-5.4]	0 [0.0-3.2]	1 (1.3) [0.2-7.2]	0 [0.0-4.8]	1 (0.7) [0.1-3.6]	1 (0.8) [0.1-4.4]	0 [0.0-2.6]	1 (0.4) [0.1-2.1]
All	8 (1.3) [0.7-2.6]	13 (2.1) [1.2-3.6]	21 (1.7) [1.1-2.6]	6 (1.1) [0.5-2.4]	13 (2.3) [1.3-3.8]	19 (1.7) [1.1-2.7]	14 (1.2) [0.7-2.0]	26 (2.2) [1.5-3.2]	40 (1.7) [1.3-2.3]

### Associations

Table 5 shows a number of sociodemographic variables and their distributions among those with and without headache; it highlights the differences between the headache types. While there were no significant differences in mean age, multivariate analysis (Table 6) shows that all headache and TTH were more prevalent in younger people. The female predilection for migraine, headache on ≥15 days/month and, especially, pMOH is clearly demonstrated in Table 5, while the AORs in Table 6 emphasise this. All specific types of headache showed an association with rural dwelling (Table 1, Table 2, Table 3, and Table 5), although this remained significant and highly so (*p* = 0.002) only for migraine in multivariate analysis (Table 6).

**Table 5:** Association of headache disorders with sociodemographic variables

Variable	No headache (n = 841)	Migraine (n = 597)	Tension-type headache (n = 811)	pMOH (n = 28)	Other headache on ≥15 d/m (n = 40)
Age (years)					
18-25	15.1 %	12.1 %	17.5 %	7.1 %	20.0 %
26-35	26.2 %	27.8 %	26.5 %	35.7 %	25.0 %
36-45	21.9 %	29.8 %	27.5 %	14.3 %	30.0 %
46-55	19.4 %	16.9 %	16.3 %	17.9 %	17.5 %
56-65	17.5 %	13.4 %	12.2 %	25.0 %	7.5 %
Mean age (SD)	39.2 (13.5)	38.1 (12.0)	36.9 (12.4)	40.8 (15.2)	35.1 (11.3)
Gender					
Male	61.8 %	35.5 %	47.8 %	14.3 %	35.0 %
Female	38.2 %	64.5 %	52.2 %	85.7 %	65.0 %
Habitation					
Rural	37.8 %	54.9 %	51.3 %	60.7 %	47.5 %
Urban	62.2 %	45.1 %	48.7 %	39.3 %	52.5 %
Household income (INR per month)					
Median	5,500	4,000	4,000	2,750	6,000
Occupation					
Professional or semi-professional	6.9 %	5.2 %	5.1 %	0.0 %	5.0 %
Clerical, shop owner, farmer	33.1 %	25.5 %	26.8 %	7.1 %	17.5 %
Skilled or semi-skilled worker	46.6 %	62.2 %	58.8 %	71.4 %	67.5 %
Unskilled worker	2.9 %	1.3 %	1.5 %	10.7 %	2.5 %
Unemployed	10.6 %	5.9 %	7.9 %	10.7 %	7.5 %
Education					
Professional or (post)graduate	16.3 %	9.3 %	12.5 %	0.0 %	10.0 %
Post-high, high or middle school	47.6 %	41.5 %	47.1 %	35.6 %	50.0 %
Primary school	7.7 %	9.0 %	7.9 %	7.1 %	5.0 %
Illiterate	28.3 %	40.0 %	32.6 %	57.1 %	35.0 %

pMOH: probable medication-overuse headache; d/m: days/month

**Table 6:** Multivariate logistic regression analysis for associations with sociodemographic variables

Variable	Any headache		Migraine		Tension-type headache		pMOH		Other headache on ≥15 d/m	
	AOR	*p*	AOR	*p*	AOR	*P*	AOR	*p*	AOR	*p*
Age 18–35 years (reference ≥36 years)	1.2	0.043	0.9	0.841	1.2	0.039	1.0	0.958	0.9	0.770
Habitation rural (reference urban)	1.8	<0.0001	1.5	0.002	1.2	0.08	2.1	0.139	1.5	0.359
Gender female (reference male)	2.3	<0.0001	2.1	<0.0001	1.1	0.548	5.9	0.001	1.9	0.065
Income ≤ INR 5,000 per month (reference >5,000)	1.1	0.452	1.1	0.718	1.1	0.287	0.712	0.492	0.5	0.12

pMOH: probable medication-overuse headache; d/m: days/month

Table 5 presents indicators of socioeconomic status. These are not easily analysed, but those with headache tended to have lower household incomes and be less well educated than those without, trends that were greatly magnified in those with pMOH. The data on employment are complex; of note only is that, among those with pMOH, none were professional and only two of 28 (7.1 %) were in the category of clerical, shop owner or farmer. There were no associations with income surviving multivariate analysis: in Table 6, we used the median income of the sample (INR 5,000/month) to create upper and lower income categories.

## Discussion

With well over one billion people [8], India is behind only China in its proportion of the world’s population (16.7 %). This study was the first large-scale community-based survey exclusively of headache in India. In other words, the findings will fill a large knowledge void not only for the country but also globally.

Over two-thirds of India’s inhabitants live in villages, the remaining 31 % in towns and urban agglomerations [8]. Our primary purpose in gathering knowledge of headache was to demonstrate the need for headache services, and this established the importance of fully representing both urban and rural populations in our survey. By the same token, this was not an easy environment in which to conduct epidemiological studies. Accordingly we invested heavily in careful methodology [14]: we had a large sample and a high participation rate (>90 %), which would have reduced the likelihood of participation bias; the survey instrument was developed after field testing in a pilot study (and has since, in various adaptations, been used with success in many other countries, cultures and languages [13]); the field investigators were rigorously trained and supervised; there was strong emphasis on quality control; we undertook a validation study in a sub sample of participants.

The observed 1 year prevalence of any headache in the study population (63.9 %), which had the usual female preponderance of close to 4:3, was in keeping with and higher than many reports from other countries [1]. In fact this statistic (1 year prevalence of any headache) is highly variable, being very susceptible to cultural tendencies reflected in the reporting (or not) of mild and/or occasional episodic headache.

The 1 year age-standardised prevalence of migraine was 25.2 %, considerably higher among females than males and higher among those from rural areas than urban. The 25.2 % is remarkable. While GBD2010 found migraine to be the third commonest disease in the world, it estimated the global prevalence at a much lower 14.7 % [3]. On the other hand, the literature review by Stovner et al. showed that reported migraine prevalence varied widely country by country [1], explicable to a large extent by methodological differences (population of interest, sampling techniques, selection of respondents, interview methods and diagnostic approach) [7] and therefore only in part by true variation (attributable to genetics, culture, lifestyle or environment). Among the now several studies supported by LTB which have employed similar methodology (including sampling technique), estimates of migraine prevalence have been 9.3 % in China [19], 20.8 % in Russia [20] and 22.9 % in Zambia [21]. All of these included both definite and probable migraine, which is methodologically correct provided that (as has always been the case) the latter have been shown not to meet criteria for definite TTH [7]. Our estimate of 25.2 % puts this southern State in India beyond this range, which is the most important finding of this study: the prevalence of migraine is very high. Because of the careful methodology and quality assurance, we believe this to be real, and largely explained in India by the latter factors referred to above: culture, lifestyle and environment. Our observed gender differential is reported almost universally. The higher prevalence in rural areas may be related to socioeconomic conditions (diet, stress and relative poverty [22, 23]) or to lack of availability and utilization of health-care facilities. The relationship between migraine prevalence and age showed an unusual second increase after age 56 years, but in the rural population only. While it was significant (*p* < 0.001) in men only, the fact that it was reflected among women suggests a real effect and, perhaps, a greater influence of those same socioeconomic influences among older people. We do not know.

Studies to elucidate the causes of this very high migraine prevalence should be given high priority, because some of them may be remediable.

There is not much to be said about TTH: the age-standardized 1 year prevalence of 35.1 % is well within the range reported from other countries [1]. The focus only on the most bothersome headache in any participant reporting more than one headache type was likely to have caused some under-reporting of TTH. This disorder showed no associations except with age: somewhat interestingly, this is a disorder more prevalent among younger people (male and female) in this country. We have no explanation for this other than to suggest that, to the extent TTH is a stress-related disorder, younger people might be more stressed in India (or older people cope better).

The estimated prevalence of all headache on ≥15 days/month was 3.0 %, equal to the global mean [1, 24], while that of pMOH was 1.2 %. Estimates of pMOH prevalence vary around the world, up to 7 % [22], but most are within 1–1.5 % [25]. The rural preponderance (which was not significant) might happen because of less easy access to health care, but no great difficulty in obtaining analgesics over-the-counter – the most common cause of MOH. What is striking is the five-fold greater prevalence among females than males. A gender difference is usual, but not one this great. We suspect it reflects a culturally-determined gender difference in health-care seeking behaviour, such that females in this part of India rely more than males on self-management and over-the-counter medication. Furthermore, because of low importance attached to headache, females, especially in rural areas, would receive little encouragement to seek health care for it. It should be remembered that the literacy rate in India is considerably lower in females (64.6 %) than males (81.0 %) [26], and this is emphasized in rural areas.

The crucial point of discussion is the issue of extrapolation: how representative are these findings of India? In truth we do not know: India is multicultural and geographically and environmentally diverse. In terms of filling the knowledge gap – for the Global Burden of Disease Study, for example – until now that gap has covered the entire South-East Asia Region! For health policy-makers in India, here are data. We recommend that at least one more study be done, in the north of the country, which may be sufficient if its findings are similar. Meanwhile, although these data are only from parts of a single State in the south of the country, they are the best information available for the entire country and its population of over 1.2 billion people [8].

## Conclusions

This was a carefully conducted study with considerable methodological strengths. It has shown a very high 1 year prevalence of migraine in south India, probably explained at least partly by cultural, lifestyle and/or environmental factors. Some of these may be remediable, a possibility that calls for further studies as a high priority. Levels of TTH, pMOH and other headache on ≥15 days/month are similar to global averages. A strong association of pMOH with female gender requires explanation. Until another study is conducted elsewhere in the country, these are the best data available to inform health policy for more than 1.2 billion people.

## References

[1] Stovner LJ, Hagen K, Jensen R, Katsarava Z, Lipton RB, Scher AI, Steiner TJ, Zwart J-A (2007). The global burden of headache: a documentation of headache prevalence and disability worldwide. Cephalalgia.

[2] World Health Organisation and Lifting The Burden (2011). ATLAS of headache disorders and resources in the world 2011.

[3] Vos T, Flaxman AD, Naghavi M, Lozano R, Michaud C, Ezzati M, Shibuya K, Salomon JA, Abdalla S, Aboyans V, Abraham J, Ackerman I, Aggarwal R, Ahn SY, Ali MK, Alvarado M, Anderson HR, Anderson LM, Andrews KG, Atkinson C, Baddour LM, Bahalim AN, Barker-Collo S, Barrero LH, Bartels DH, Basáñez MG, Baxter A, Bell ML, Benjamin EJ, Bennett D (2012). Years lived with disability (YLDs) for 1160 sequelae of 289 diseases and injuries 1990–2010: a systematic analysis for the Global Burden of Disease Study 2010. Lancet.

[4] Gourie-Devi M, Gururaj G, Satishchandra P, Subbakrishna DK (2004). Prevalence of neurological disorders in Bangalore, India: a community-based study with a comparison between urban and rural areas. Neuroepidemiology.

[5] Ravishankar K, Chakravarthy A (2002). Headache – the Indian experience. Annals Indian Acad Neurol.

[6] Panda S, Tripathi M (2005). Clinical profile of migraineurs in a referral centre in India. J Assoc Physicians India.

[7] Stovner LJ, Al Jumah M, Birbeck GL, Gururaj G, Jensen R, Katsarava Z, Queiroz LP, Scher AI, Tekle-Haimanot R, Wang SJ, Steiner TJ (2014). The methodology of population surveys of headache prevalence, burden and cost: principles and recommendations from the Global Campaign against Headache. J Headache Pain.

[8] Government of India (2011) 2011 census data. Available at http://censusindia.gov.in/ (last accessed 30 April 2015).

[9] Steiner TJ (2004). Lifting the burden: the global campaign against headache. Lancet Neurol.

[10] Steiner TJ (2005). *Lifting The Burden*: the global campaign to reduce the burden of headache worldwide. J Headache Pain.

[11] Steiner TJ, Birbeck GL, Jensen R, Katsarava Z, Martelletti P, Stovner LJ (2010). *Lifting The Burden*: the first 7 years. J Headache Pain.

[12] Steiner TJ, Birbeck GL, Jensen R, Katsarava Z, Martelletti P, Stovner LJ (2011). The Global Campaign, World Health Organization and *Lifting The Burden*: collaboration in action. J Headache Pain.

[13] Steiner TJ, Gururaj G, Andrée C, Katsarava Z, Ayzenberg I, Yu S-Y, Al Jumah M, Tekle-Haimanot R, Birbeck GL, Herekar A, Linde M, Mbewe E, Manandhar K, Risal A, Jensen R, Queiroz LP, Scher AI, Wang SJ, Stovner LJ (2014). Diagnosis, prevalence estimation and burden measurement in population surveys of headache: presenting the HARDSHIP questionnaire. J Headache Pain.

[14] Rao GN, Kulkarni GB, Gururaj G, Rajesh K, Subbakrishna DK, Steiner TJ, Stovner LJ (2012). The burden of headache disorders in India: methodology and questionnaire validation for a community-based survey in Karnataka State. J Headache Pain.

[15] Peters M, Bertolote JM, Houchin C, Kandoura T, Steiner TJ (2007). Translation protocol for hybrid documents. J Headache Pain.

[16] Headache Classification Subcommittee of the International Headache Society (2004). The International Classification of Headache Disorders: 2nd edition. Cephalalgia.

[17] Dean AG, Arner TG, Sunki GG, Friedman R, Lantinga M, Sangam S, Zubieta JC, Sullivan KM, Brendel KA, Gao Z, Fontaine N, Shu M, Fuller G, Smith DC, Nitschke DA, Fagan RF (2007). Epi Info™, a database and statistics program for public health professionals, version 3.5.1.

[18] SPSS Inc (2006). Statistical package for social sciences, USA version 15.0.

[19] Yu S, Liu R, Zhao G, Yang X, Qiao X, Feng J, Fang Y, Cao X, He M, Steiner T (2012). The prevalence and burden of primary headaches in China: a population-based door-to-door survey. Headache.

[20] Ayzenberg I, Katsarava Z, Sborowski A, Chernysh M, Osipova V, Tabeeva G, Yakhno N, Steiner TJ (2012). The prevalence of primary headache disorders in Russia: a countrywide survey. Cephalalgia.

[21] Mbewe E, Zairemthiama P, Paul R, Yeh H-H, Birbeck GL, Steiner TJ (2015). The epidemiology of primary headache disorders in Zambia: a population-based door-to-door survey. J Headache Pain.

[22] Fernandez-de-Las-Penas C, Hernandez-Barrera V, Carrasco-Garrido P, Alonso-Blanco C, Palacios-Cena D, Jimenez-Sanchez S, Jimenez-Garcia R (2010). Population-based study of migraine in Spanish adults: relation to socio-demographic factors, lifestyle and co-morbidity with other conditions. J Headache Pain.

[23] Winter AC, Berger K, Buring JE, Kurth T (2012). Associations of socioeconomic status with migraine and non migraine headache. Cephalalgia.

[24] Jensen R, Stovner LJ (2008). Epidemiology and comorbidity of headache. Lancet Neurol.

[25] Westergaard ML, Hansen EH, Glumer C, Olesen J, Jensen RH (2014). Definitions of medication-overuse headache in population-based studies and their implications on prevalence estimates: a systematic review. Cephalalgia.

[26] Government of India (2013) Poverty estimates 2011–12 of Planning Commission. Available at http://planningcommission.nic.in/news/pre_pov2307.pdf (last accessed 7 May 2015).

